# Diverse Regulatory Manners and Potential Roles of lncRNAs in the Developmental Process of Asian Honey Bee (*Apis cerana*) Larval Guts

**DOI:** 10.3390/ijms242015399

**Published:** 2023-10-20

**Authors:** Xiaoxue Fan, Xuze Gao, He Zang, Sijia Guo, Xin Jing, Yiqiong Zhang, Xiaoyu Liu, Peiyuan Zou, Mengjun Chen, Zhijian Huang, Dafu Chen, Rui Guo

**Affiliations:** 1 College of Animal Sciences (College of Bee Science), Fujian Agriculture and Forestry University, Fuzhou 350002, China; imfanxx@163.com (X.F.); gxz13845381765@163.com (X.G.); zanghe321@163.com (H.Z.); guosijia1998@163.com (S.G.); jingxin6662022@163.com (X.J.); zhangyiqiong1121@163.com (Y.Z.); liuxiaoyu2000@163.com (X.L.); zoupeiyuan2216@163.com (P.Z.); w532816764@163.com (M.C.); 18350038128@163.com (Z.H.); dfchen826@fafu.edu.cn (D.C.); 2 Apitherapy Research Institute of Fujian Province, Fuzhou 350002, China

**Keywords:** non-coding RNA, lncRNA, honey bee, *Apis cerana cerana*, gut, development, regulation

## Abstract

Long non-coding RNAs (lncRNAs) are crucial modulators in a variety of biological processes, such as gene expression, development, and immune defense. However, little is known about the function of lncRNAs in the development of Asian honey bee (*Apis cerana*) larval guts. Here, on the basis of our previously obtained deep-sequencing data from the 4-, 5-, and 6-day-old larval guts of *A. cerana* workers (Ac4, Ac5, and Ac6 groups), an in-depth transcriptome-wide investigation was conducted to decipher the expression pattern, regulatory manners, and potential roles of lncRNAs during the developmental process of *A. cerana* worker larval guts, followed by the verification of the relative expression of differentially expressed lncRNAs (DElncRNAs) and the targeting relationships within a competing endogenous RNA (ceRNA) axis. In the Ac4 vs. Ac5 and Ac5 vs. Ac6 comparison groups, 527 and 498 DElncRNAs were identified, respectively, which is suggestive of the dynamic expression of lncRNAs during the developmental process of larval guts. A *cis*-acting analysis showed that 330 and 393 neighboring genes of the aforementioned DElncRNAs were respectively involved in 29 and 32 functional terms, such as cellular processes and metabolic processes; these neighboring genes were also respectively engaged in 246 and 246 pathways such as the Hedgehog signaling pathway and the Wnt signaling pathway. Additionally, it was found that 79 and 76 DElncRNAs as potential antisense lncRNAs may, respectively, interact with 72 and 60 sense-strand mRNAs. An investigation of competing endogenous RNA (ceRNA) networks suggested that 75 (155) DElncRNAs in the Ac4 vs. Ac5 (Ac5 vs. Ac6) comparison group could target 7 (5) DEmiRNAs and further bind to 334 (248) DEmRNAs, which can be annotated to 33 (29) functional terms and 186 (210) pathways, including 12 (16) cellular- and humoral-immune pathways (lysosome pathway, necroptosis, MAPK signaling pathway, etc.) and 11 (10) development-associated signaling pathways (Wnt, Hippo, AMPK, etc.). The RT-qPCR detection of five randomly selected DElncRNAs confirmed the reliability of the used sequencing data. Moreover, the results of a dual-luciferase reporter assay were indicative of the binding relationship between MSTRG.11294.1 and miR-6001-y and between miR-6001-y and ncbi_107992440. These results demonstrate that DElncRNAs are likely to modulate the developmental process of larval guts via the regulation of the source genes’ transcription, interaction with mRNAs, and ceRNA networks. Our findings not only yield new insights into the developmental mechanism underlying *A. cerana* larval guts, but also provide a candidate ceRNA axis for further functional dissection.

## 1. Introduction

Non-coding RNAs (ncRNAs), generally used to indicate RNA molecules that do not encode proteins, have recently been recognized as important regulators of biological systems [1]. Different types of ncRNAs are transcribed from the genome and perform their respective biological functions at the RNA level [2]. NcRNAs can be arbitrarily categorized into two types according to their sizes: one type comprises small RNAs with a length shorter than 200 nt; and the other type comprises long non-coding RNAs (lncRNAs) [3], a class of linear RNA molecules transcribed by RNA polymerase II or RNA polymerase III [4]. LncRNAs are usually expressed at low levels, lack conservation among species, and display tissue- or cell-specific expression patterns [5,6]. The accumulating evidence indicates that lncRNAs are capable of exerting critical functions in the regulation of numerous biological processes in multiple manners, such as through a *cis*-acting effect or a competing endogenous RNA (ceRNA) mechanism [7,8].

*Apis cerana* is a major bee species distributed in eastern, southern, and southeastern Asia and is widely used in beekeeping practices [9]. *A. cerana* has a subseries of advantages, such as a high resistance to low temperatures, a great ability to collect sporadic nectar sources, a long honey-collection period, and a strong resilience to ectoparasitic mites [10,11,12]. As the nominate subspecies of *A. cerana*, *Apis cerana cerana* is reared in China and many other Asian countries, with great ecological and economical value [13].

In insects, lncRNAs are suggested to participate in the regulation of the embryo development, neurological function, gonadal function, and anti-stress ability of *Drosophila* [14]; the innate immune of the red flour beetle (*Tribolium castaneum*) [15]; and the alternative splicing of the *Bmdsx* gene in silkworms (*Bombycis mori*) [16]. For instance, Chen et al. [17] identified abundant lncRNAs in the embryos, eggs, nymphs, and adults of *Sogatella furcifera*; Chang et al. [18] found a high proportion of up-regulated lncRNAs in embryonic, fourth-instar, and fifth-instar nymphs. A suite of studies has shown that lncRNAs play pivotal roles in the growth [19], development [20], caste differentiation [21], and innate immunity [22] of honey bees. Huang et al. [19] exposed *Apis mellifera* to 0.01 mg/L of dinotefuran for 1, 5, or 10 d and performed lncRNA sequencing on the dinotefuran-treated group. The results showed that lncRNAs were involved in the Hippo and transforming growth factor-β (TGF-β) signaling pathways. However, relevant studies on *A. cerana* lncRNAs were limited. Following the investigation of the expression pattern and regulatory manner of lncRNAs, our group previously deciphered the responses of *A. cerana* to infections by *Ascosphaera apis* and *Nosema ceranae*, two widespread fungal bee pathogens [23,24].

The insect gut is a vital tissue responsible for food digestion, nutrient absorption, and immune defense [25]. In honey bees, previous studies have mainly focused on intestinal microorganisms [26,27,28], and few advancements in the developmental mechanisms of bee guts have been gained until now. In recent years, on the basis of deep sequencing and bioinformatics, we systematically analyzed the differential expression profiles and putative regulatory roles of piRNAs and miRNAs in the developmental process of *A. cerana* worker larval guts [29,30]. In this current work, the expression pattern of lncRNAs during the developmental process of the guts of *A. c. cerana* worker larvae was surveyed based on our previously gained high-quality transcriptome data, followed by an in-depth investigation of the regulatory manners and potential roles of DElncRNAs in the larval guts. Our findings not only offer a new perspective into the development of the *A. c. cerana* worker larval guts but also lay the foundation for illustrating the DElncRNA-modulated mechanisms underlying larval gut development.

## 2. Results

### 2.1. Expression Pattern of lncRNAs during the Developmental Process of Larval Guts

**Figure 1:**
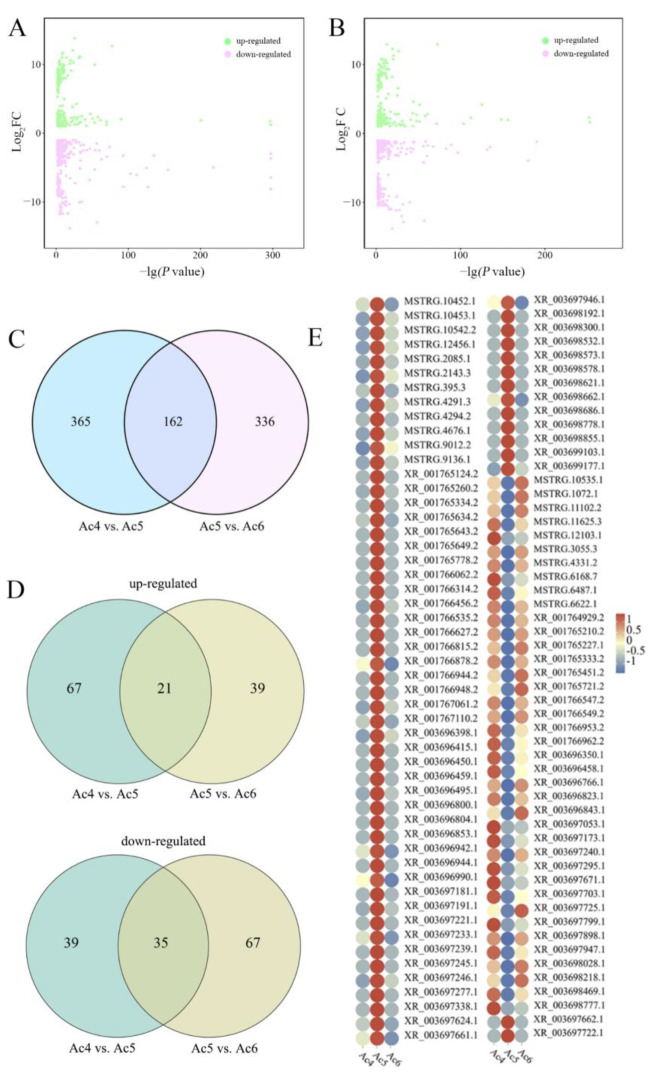
Differential analysis of lncRNAs in the guts of *A. c. cerana* worker larvae. (**A**,**B**) Manhattan maps of DElncRNAs in the Ac4 vs. Ac5 and Ac5 vs. Ac6 comparison groups; green dots indicate up-regulated lncRNAs, while pink dots indicate down-regulated lncRNAs. (**C**,**D**) Venn diagrams of up-regulated and down-regulated lncRNAs in the two comparison groups. (**E**) Heatmaps of expression clustering for the DElncRNAs shared by the two comparison groups.

In the Ac4 vs. Ac5 comparison group, 527 DElncRNAs were screened, including 215 up-regulated and 312 down-regulated lncRNAs (Figure 1A). Among these, the most up-regulated lnRNA was XR_003698855.1 (log_2_FC = 13.79), followed by MSTRG.4294.2 (log_2_FC = 12.68) and MSTRG.9012.2 (log_2_FC = 12.04), while the three most down-regulated lncRNAs, in order from most to least affected, were XR_001766962.2 (log_2_FC = −13.82), MSTRG.3055.3 (log_2_FC = −12.93), and MSTRG.9620.1 (log_2_FC = −11.76) (Figure 1A). As shown in Figure 1B, 498 DElncRNAs in the Ac5 vs. Ac6 comparison group were detected, including 203 up-regulated and 295 down-regulated lncRNAs. Among these, the three most up-regulated lncRNAs, in order from most to least affected, were MSTRG.3055.3 (log_2_FC = 12.92), XR_001766962.2 (log_2_FC = 12.91), and XR_001766735.2 (log_2_FC = 12.25), whereas the most down-regulated lncRNA was XR_003698855.1 (log_2_FC = −13.79), followed by MSTRG.7106.3 (log_2_FC = −12.86) and MSTRG.4294.2 (log_2_FC = −12.68) (Figure 1B). In addition, 148 up-regulated and 176 down-regulated lncRNAs were found to be shared by the two aforementioned comparison groups (Figure 1C,D). Moreover, it was observed that the these shared DElncRNAs displayed various expression trends during the developmental process of larval guts (Figure 1E).

### 2.2. Cis-Acting Effect of DElncRNAs on the Developmental Process of Larval Guts

**Figure 2:**
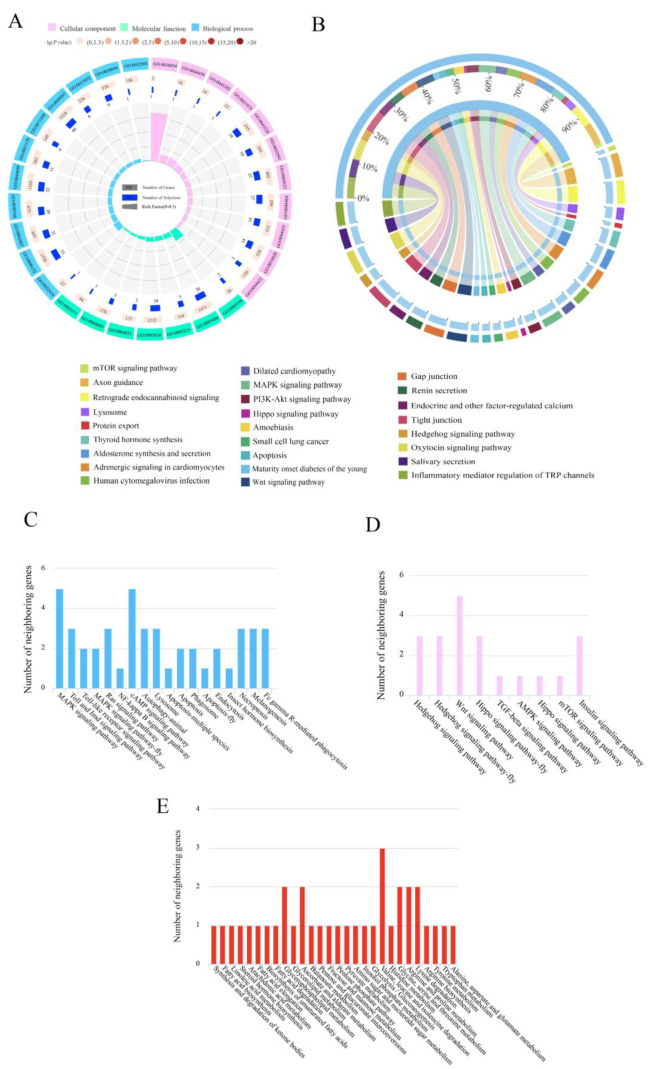
GO terms and KEGG pathways enriched by neighboring genes of DElncRNAs in the Ac4 vs. Ac5 comparison group. (**A**) Loop graph of GO terms enriched by neighboring genes. (**B**) Circos graph of KEGG pathways enriched by neighboring genes. (**C**) Number statistics of neighboring genes relevant to cellular and humoral pathways. (**D**) Number statistics of neighboring genes relevant to development-associated signaling pathways. (**E**) Number statistics of neighboring genes relevant to material-metabolism-related pathways.

Here, we investigated the cis-regulatory lncRNAs by screening the protein-coding genes as potential targets in the regions located 10 kb upstream/downstream of DElncRNAs. In the Ac4 vs. Ac5 comparison group, 239 DElncRNAs were predicted to regulate 330 neighboring genes, which were enriched in 29 GO terms related to biological processes, cellular components, and molecular functions, such as metabolic processes, cells, and binding (Figure 2A, see also Table S1). These neighboring genes were also involved in 246 KEGG pathways, including the inflammatory mediator regulation of TRP channels, the oxytocin signaling pathway, and tight junctions (Figure 2B, see also Table S1). A further investigation showed that 23 neighboring genes were enriched in 18 pathways relevant to 11 cellular and seven humoral pathways, such as the apoptosis as well as the Toll and Imd signaling pathways (Figure 2C); 10 neighboring genes were enriched in nine development-associated signaling pathways, such as the Hedgehog and Hippo signaling pathways (Figure 2D); and 17 neighboring genes were enriched in 28 material-metabolism-related pathways, such as fatty acid biosynthesis and lysine degradation (Figure 2E).

**Figure 3:**
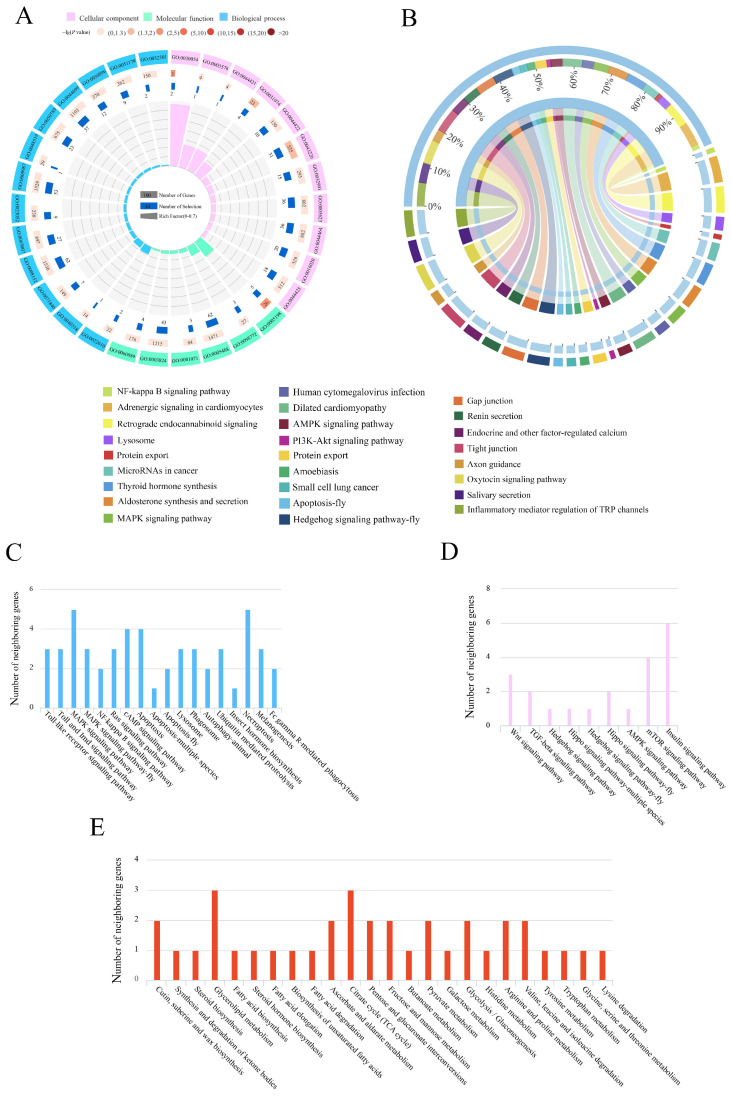
GO terms and KEGG pathways enriched by neighboring genes of DElncRNAs in the Ac5 vs. Ac6 comparison group. (**A**) GO terms enriched by neighboring genes. (**B**) KEGG pathways enriched by neighboring genes. (**C**) Number of neighboring genes that were enriched in cellular and humoral pathways. (**D**) Number of neighboring genes that were enriched in development-associated signaling pathways. (**E**) Number of neighboring genes that were enriched in material-metabolism-related pathways.

Comparatively, 266 DElncRNAs in the Ac5 vs. Ac6 comparison group were predicted to regulate 393 neighboring genes, which were involved in 32 GO terms, including metabolic processes, cell parts, and catalytic activity (Figure 3A, see also Table S2). As shown in Figure 3B, these neighboring genes were also engaged in 250 KEGG pathways, such as the PI3K-Akt signaling pathway, MAPK signaling pathway, and Wnt signaling pathway. Moreover, it was found that 29 neighboring genes were enriched in 18 pathways relevant to 11 cellular- and seven humoral-immune pathways, such as the melanogenesis and cAMP signaling pathways (Figure 3C); 13 neighboring genes were enriched in nine development-associated signaling pathways, such as the mTOR and Wnt signaling pathways (Figure 3D); and 16 neighboring genes were enriched in 24 material-metabolism-related pathways, such as glycerolipid metabolism and tryptophan metabolism (Figure 3E).

### 2.3. DElncRNAs as Antisense lncRNAs in Larval Guts

**Figure 4:**
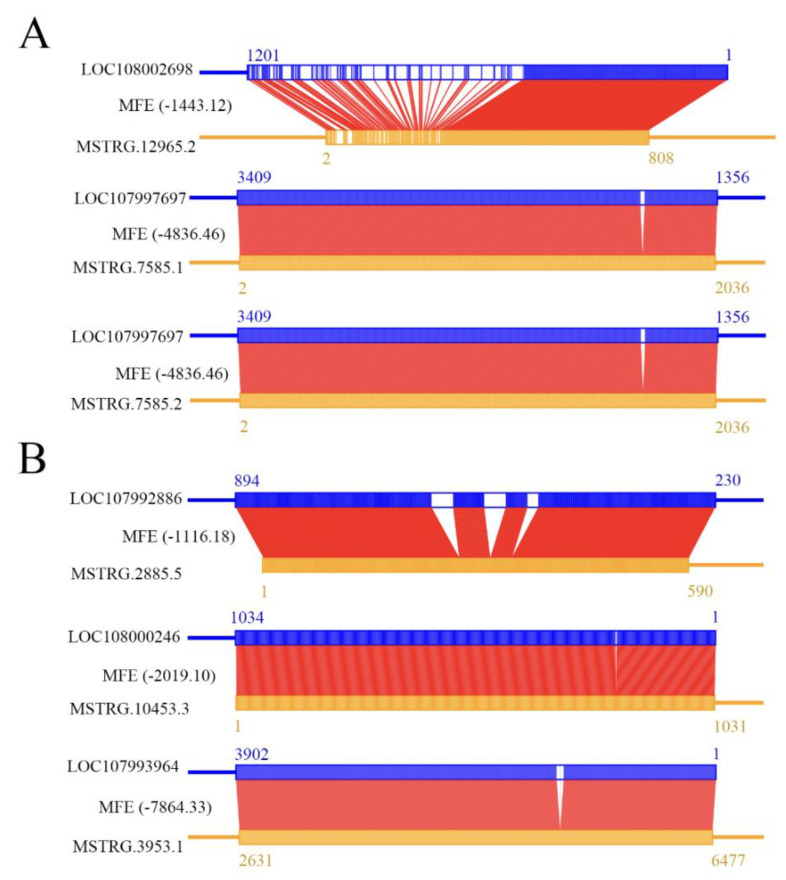
Binding relationships between six antisense DElncRNAs and corresponding sense-strand mRNAs. (**A**) Binding relationships between three DElncRNAs (MSTRG.12965.2, MSTRG.7585.1, and MSTRG.7585.2) and corresponding sense-strand mRNAs in the Ac4 vs. Ac5 comparison group. (**B**) Binding relationships between three DElncRNAs (MSTRG.2885.5, MSTRG.10453.3, and MSTRG.3953.1) and corresponding sense-strand mRNAs in the Ac5 vs. Ac6 comparison group.

Here, it was detected that 79 potential antisense DElncRNAs in the Ac4 vs. Ac5 comparison group may interact with 72 sense-strand mRNAs; e.g., both MSTRG.7585.1 and MSTRG.7585.2 may interact with the mRNA of the ecdysone-inducible protein 75 isomer X2 gene (LOC107997697), and MSTRG.12965.2 may interact with the mRNA of the insulin-like peptide receptor gene (LOC108002698) (Figure 4A). In contrast, 76 DElncRNAs in the Ac5 vs. Ac6 comparison group were predicted to be putative antisense DElncRNAs, which may interact with 60 sense-strand mRNAs; For example, the lncRNA MSTRG.2885.5 may interact with the mRNA of the cell wall integrity and stress response component 1-like (LOC107992886) gene, MSTRG.10453.3 may interact with the mRNA of homeotic protein labial-like partial (LOC108000246), and MSTRG.3953.1 may interact with the mRNA of insulin-like growth factor 2 mRNA-binding protein 1 isoform X1 (LOC107993964) (Figure 4B).

### 2.4. DElncRNA-Mediated ceRNA Regulatory Networks in the Developmental Process of Larval Guts

A ceRNA network analysis demonstrated that 75 DElncRNAs in the Ac4 vs. Ac5 comparison group could target seven DEmiRNAs and further bind to 334 DEmRNAs (Figure S1A); these targets were engaged in 33 GO terms, such as membrane parts and the regulation of biological processes, as well as 186 KEGG pathways, such as the Hippo and MAPK signaling pathways (Table S3). Additionally, 155 DElncRNAs in the Ac5 vs. Ac6 comparison group could target five DEmiRNAs, further linking to 248 DEmRNAs (Figure S1B); these targets were involved in 29 GO terms, including membrane and cellular processes, as well as 210 KEGG pathways, including the Wnt and Ras signaling pathways (Table S4). The DElncRNA–DEmiRNA and DEmiRNA–DEmRNA binding relationships are presented in Tables S5 and S6.

### 2.5. Sub-Networks Associated with Cellular and Humoral Immunity

**Figure 5:**
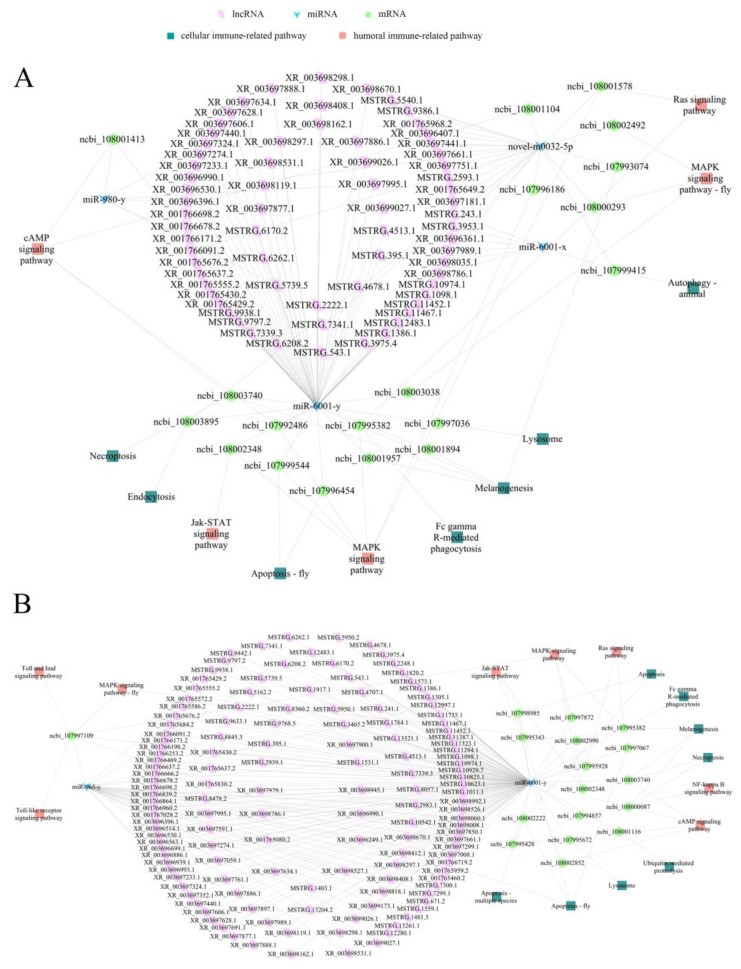
DElncRNA–DEmiRNA–DEmRNA networks relevant to cellular- and humoral-immune-related pathways. (**A**) Ac4 vs. Ac5 comparison group. (**B**) Ac5 vs. Ac6 comparison group.

A further analysis suggested that 68 DElncRNAs in the Ac4 vs. Ac5 comparison group could target four DEmiRNAs and further link to 19 DEmRNAs related to seven cellular-immune pathways (lysosome, necroptosis, melanogenesis, etc.) and five humoral-immune pathways (cAMP pathway, Ras pathway, MAPK signaling pathway, etc.) (Figure 5A, see also Table S7). In the Ac5 vs. Ac6 comparison group, 141 DElncRNAs could target two DEmiRNAs, further targeting 17 DEmRNAs associated with eight cellular-immune pathways (apoptosis, ubiquitin-mediated proteolysis, Fc gamma R-mediated phagocytosis, etc.) and eight humoral-immune pathways (Jak-STAT pathway, Toll and Imd pathways, Toll-like receptor signaling pathway, etc.) (Figure 5B, see also Table S7).

### 2.6. Sub-Networks Relevant to Development-Associated Signaling Pathways

**Figure 6:**
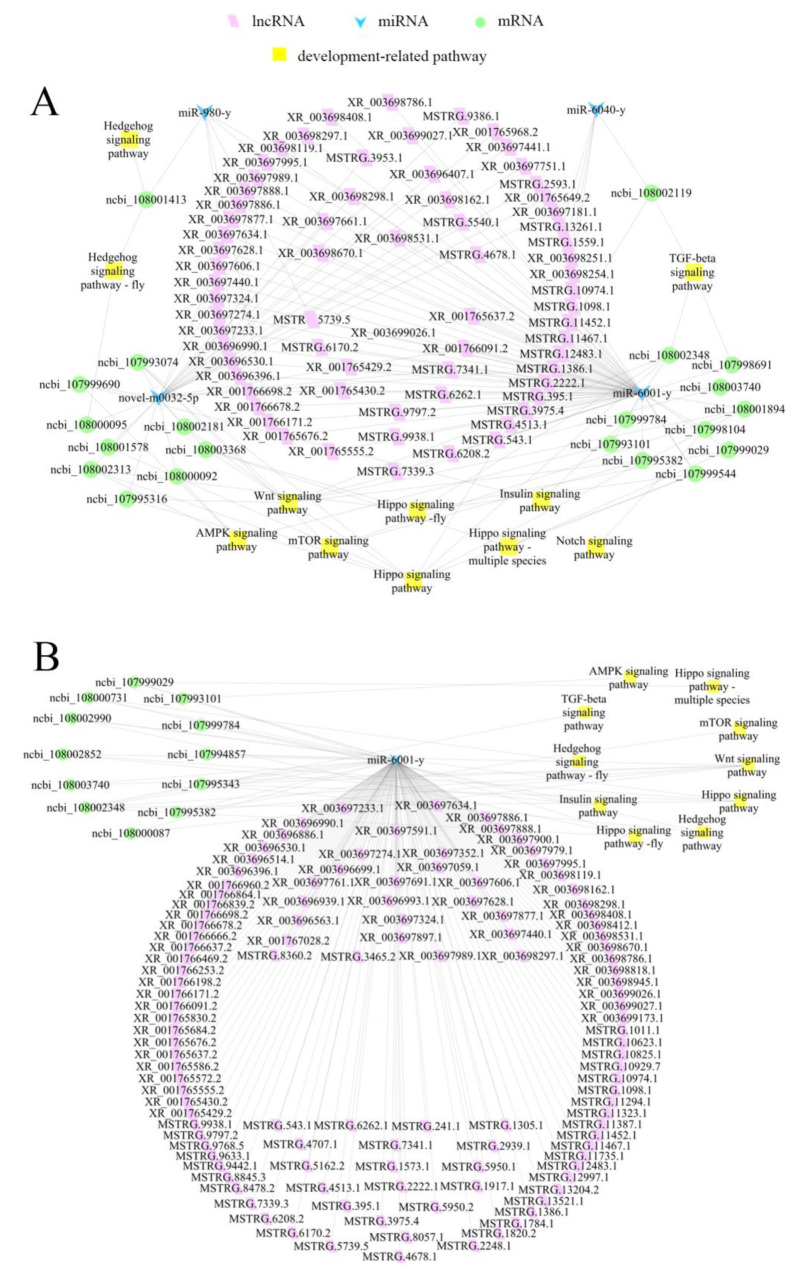
DElncRNA–DEmiRNA–DEmRNA networks relevant to development-associated signaling pathways. (**A**) Ac4 vs. Ac5 comparison group. (**B**) Ac5 vs. Ac6 comparison group.

Additionally, it was observed that 69 DElncRNAs in the Ac4 vs. Ac5 comparison group could target four DEmiRNAs and further bind to 21 DEmRNAs associated with 11 development-related signaling pathways, such as the Hippo, Wnt, and Notch signaling pathways (Figure 6A, see also Table S8).

In contrast, 116 DElncRNAs in the Ac5 vs. Ac6 comparison group could target only one DEmiRNA (miR-6001-y), further targeting 12 DEmRNAs related to 10 development-relevant signaling pathways, such as the Hedgehog, TGF-beta, and mTOR signaling pathways (Figure 6B, see also Table S8).

### 2.7. RT-qPCR Confirmation of DElncRNAs

**Figure 7:**
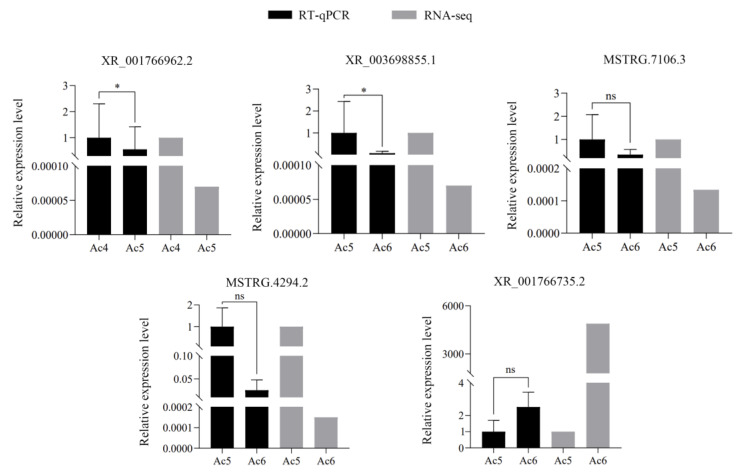
RT-qPCR detection of five DElncRNAs. Data are presented as means ± standard deviation (SD) and were subjected to Student’s *t*-test; ns, *p* > 0.05; *, *p* < 0.05.

Here, five DElncRNAs were randomly selected for RT-qPCR verification, and the results demonstrated that the expression trends of these DElncRNAs were consistent with those in the transcriptome data (Figure 7), which indicated that the sequencing data used in this work were authentic and reliable.

### 2.8. Verification of the Binding Relationship between MSTRG.11294.1 and miR-6001-y and between miR-6001-y and ncbi_107992440

**Figure 8:**
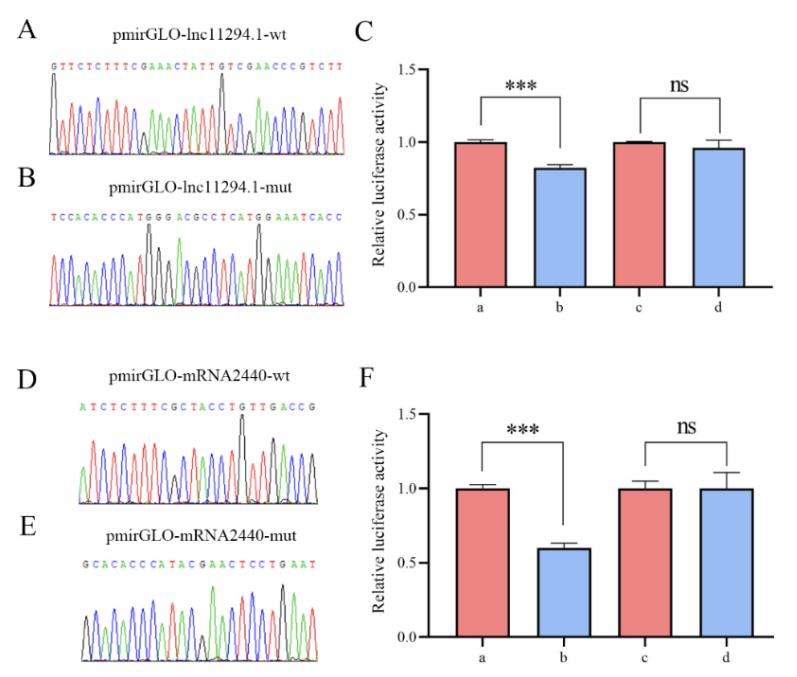
Validation of the binding relationships between MSTRG.11294.1 and miR-6001-y and between miR-6001-y and ncbi_107992440. (**A**,**B**) Sanger sequencing results of the amplified binding site from the pmirGLO-lnc11294.1-wt and the mutated binding site from the pmirGLO-lnc11294.1-mut. (**C**) Dual-luciferase reporter assay of the binding relationship between MSTRG.11294.1 and miR-6001-y. a: Co-transfected cells with mimic-NC and pmirGLO-lnc11294.1-wt; b: co-transfected cells with mimic-miR-6001-y and pmirGLO-lnc11294.1-wt; c: co-transfected cells with Mimic-NC and pmirGLO-lnc11294.1-mut; and d: co-transfected cells with Mimic-miR-6001-y and pmirGLO-lnc11294.1-mut. (**D**,**E**) Sanger sequencing results for the amplified binding sites and mutated binding sites of the pmirGLO-mRNA2440-wt and pmirGLO-mRNA2440-mut plasmids. (**F**): Dual-luciferase reporter assay of the binding relationship between miR-6001-y and ncbi_107992440. a: Co-transfected cells with Mimic-NC and pmirGLO-mRNA2440-wt; b: co-transfected cells with mimic-miR-6001-y and pmirGLO-mRNA2440-wt; c: co-transfected cells with mimic-NC and pmirGLO-mRNA2440-mut; and d: co-transfected cells with Mimic-miR-6001-y and pmirGLO-mRNA2440-mut. Data are presented as means ± standard deviation (SD) and were subjected to Student’s *t*-test; ns, *p* > 0.05; ***, *p* < 0.001.

The Sanger sequencing results verified that the recombinant plasmids pmirGLO-lnc11294.1-wt, pmirGLO-lnc11294.1-mut, pmirGLO-mRNA2440-wt, and pmirGLO-mRNA2440-mut were successfully constructed (Figure 8A, B, D, E). The dual luciferase assay suggested that the cell fluorescence activity of the mimic-miR-6001-y and pmirGLO-lnc11294.1-wt co-transfected group was significantly reduced (*p* < 0.001) in comparison to that of the Mimic-NC and pmirGLO-lnc11294.1-wt co-transfected group, whereas a non-significant change (*p* > 0.05) in the cell fluorescence activity was detected between the Mimic-mic-6001-y and pmirGLO-lnc11294.1-mut co-transfected group and the Mimic-NC and pmirGLO-lnc11294.1-mut co-transfected group. Similarly, when compared with that of the Mimic-NC and pmirGLO-mRNA2440-wt co-transfected group, the cell fluorescence activity of the mimic-miR-6001-y and pmirGLO-mRNA2440-wt co-transfected group was significantly reduced (*p* < 0.001) (Figure 8C), while a non-significant alteration (*p* > 0.05) in cell fluorescence activity was observed between the mimic-miR-6001-y and pmirGLO-mRNA2440-mut co-transfection group and the mimic-NC and pmirGLO-mRNA2440-mut co-transfection group (Figure 8F). These results confirmed the binding relationships between MSTRG.11294.1 and miR-6001-y as well as between miR-6001-y and ncbi_107992440.

## 3. Discussion

### 3.1. The Developmental Process of A. c. Cerana Worker Larval Guts Was Accompanied by a Dynamic Alteration in lncRNAs

Previous studies have shown that the overall expression profile of lnRNAs changes during the developmental process of the different organs and tissues of various insects [1,31,32]. In this study, 215 up-regulated and 312 down-regulated lncRNAs were detected in the Ac4 vs. Ac5 comparison group, while 203 up-regulated and 295 down-regulated lncRNAs were observed in the Ac5 vs. Ac6 comparison group. A total of 148 up-regulated and 176 down-regulated ones were shared (Figure 1), which is indicative of the dynamic alteration in the expression pattern of lncRNAs during the developmental process of *A. c. cerana* worker larval guts. This implies that these DElncRNAs are putative regulators in the development of larval guts. Our previous work suggested that miRNAs and piRNAs are differentially expressed and potentially participate in the developmental processes of the larval guts of *A. c. cerana* workers. Collectively, these results demonstrate that various types of ncRNAs, including lncRNAs, miRNAs, and piRNAs, are likely to participate in the modulation of larval gut development through single regulation or co-regulation via cross-talk, as verified in previous studies [20,29]. A further analysis showed that the expression levels of several lncRNAs were continuously elevated with an increase in the developmental time, which is suggestive of their pivotal roles in the developmental process, thus deserving further functional investigations.

### 3.2. DElncRNAs Were Likely to Participate in the Modulation of the Developmental Process of Larval Guts in a Cis-Acting Manner

LncRNAs are able to regulate the transcription of neighboring genes and further exert specific functions [33,34,35]. In *Aedes albopictus*, 13 DElncRNAs potentially regulated genes involved in immune-related functions, such as the innate immune response, in a *cis*-acting manner [36]. The gut tissue of honey bees is the major site for material metabolisms [25]. Here, in the Ac4 vs. Ac5 and Ac5 vs. Ac6 comparison groups, the neighboring genes of DElncRNAs were, respectively involved in 28 and 24 metabolism-related pathways of great importance, such as fatty acid biosynthesis, lysine degradation, glycerolipid metabolism, and tryptophan metabolism (Figure 2E and Figure 3E), which indicated that the corresponding DElncRNAs were likely to extensively participate in the regulation of material metabolisms in the larval guts.

The development of the insect gut is a complex process regulated by multiple signaling pathways, such as the Hippo, Wnt, and Hedgehog pathways [37,38,39]. The Hippo signaling pathway can not only regulate organ size by inhibiting cell proliferation and promoting apoptosis [40,41] but also interact with other signaling pathways to modulate the homeostasis of the midgut tissue [42]. In the present study, two DElncRNAs (MSTRG.7039.1 and MSTRG.7039.3) in the Ac4 vs. Ac5 comparison group potentially regulated the transcription of the enriched ncbi_107997224 gene in the Hippo signaling pathway (Figure 2D). The Wnt signaling pathway is closely related to a subseries of physiological processes, such as mammal embryogenesis, ovary development, and planar cell polarity [43]; it is also able to affect somitogenesis, pigmentation, and the development of organs in conjunction with the Hippo, Notch, and TGF-beta signaling pathways [44]. Here, five and three neighboring genes of six and four DElncRNAs, respectively, were involved in the Wnt signaling pathway; for example, MSTRG.6910.1 in the Ac4 vs. Ac5 comparison group putatively regulated the transcription of the ncbi_107997013 gene, and XR_003696838.1 in the Ac5 vs. Ac6 comparison group putatively regulated the transcription of the ncbi_107994517 gene (Figure 2D and Figure 3D). In *Drosophila*, the Hedgehog signaling pathway has been suggested to play a vital part in the modulation of processes from cell growth and wing morphology to germ cell migration and the development of the lymph gland and intestine [45,46,47,48]. In this work, three and one neighboring genes of four and two DElncRNAs, respectively, were engaged in the Hedgehog signaling pathway; for example, MSTRG.7987.3 in the Ac4 vs. Ac5 comparison group putatively regulated the transcription of the ncbi_107998235 gene, and XR_003697644.1 in the Ac5 vs. Ac6 comparison group putatively regulated the transcription of the ncbi_107997548 gene (Figure 2D and Figure 3D). In summary, these results indicated that the corresponding DElncRNAs might regulate the Hippo, Wnt, and Hedgehog signaling pathways during the developmental process of larval guts in a *cis*-acting way.

Insects, including honey bees, only possess an innate immune system, which can be divided into humoral and cellular immunity and is utilized by insects to defend against various pathogens and parasites [49]. Here, the neighboring genes of DElncRNAs in the Ac4 vs. Ac5 comparison group were involved in 11 cellular-immune pathways, such as apoptosis and endocytosis, and seven humoral-immune pathways, such as the Toll, Imd, and MAPK signaling pathways; the neighboring genes of DElncRNAs in the Ac5 vs. Ac6 comparison group were engaged in 11 cellular-immune pathways (e.g., lysosome- and ubiquitin-mediated proteolysis) and seven humoral-immune pathways (e.g., the Toll-like receptor and cAMP signaling pathways). Additionally, 17 immune pathways, such as the lysosome pathway, phagosome pathway, apoptosis pathway, MAPK signaling pathway, Ras signaling pathway, and cAMP signaling pathway, were enriched by the neighboring genes of DElncRNAs (XR_001766815.2, MSTRG.7339.3, XR_001765227.1, etc.) in both comparison groups (Figure 2C and Figure 3C). Collectively, the results showed that the corresponding DElncRNAs were potentially involved in the modulation of the cellular and humoral immunity of the larval guts through the regulation of the transcription of neighboring genes.

### 3.3. Antisense DElnRNAs Might Interact with Sense mRNAs to Regulate the Developmental Process of Larval Guts

In addition to the *cis*-action, lncRNAs could also act as antisense lnRNAs that complementarily pair with sense mRNAs to regulate their stability and translation [4,34,35]. In insects, 20-hydroxyecdysone (20E) and juvenile hormone (JH) have been suggested to antagonistically act to orchestrate life-history traits, including growth, development, and reproduction [50,51]. In this current work, two DElncRNAs (MSTRG.7585.1 and MSTRG.7585.2) in the Ac4 vs. Ac5 comparison group were found to putatively target the mRNA of the gene encoding ecdysone-inducible protein 75 isomer X2 (LOC107997697) (Figure 4A), indicating the potential participation of MSTRG.7585.1 and MSTRG.7585.2 in the regulation of larval guts by acting as antisense lncRNAs.

The insulin-like peptide receptor is present in all multicellular organisms, and it is capable of regulating diverse processes such as the growth, development, metabolic homeostasis, and lifespan of *Drosophila* [52,53]. Here, MSTRG.12965.2 (log_2_FC = −2.53, *p* = 2.5 × 10^−5^) in the Ac4 vs. Ac5 comparison group could target the mRNA of the gene encoding the insulin-like peptide receptor (Figure 4A), implying the involvement of MSTRG.12965.2 in the regulation of the expression of the insulin-like peptide receptor gene during the developmental process of larval guts. The cell wall is a rigid cellular structure required for the maintenance of the cell shape and protection against environmental stresses. During growth and morphogenesis, and the environmental stress response to *Saccharomyces cerevisiae*, the cell wall is remodeled in a highly regulated and polarized manner, a process that is principally under the control of the cell wall integrity signaling pathway [54]. We observed that MSTRG.2885.5, a putative antisense lncRNA in the Ac5 vs. Ac6 comparison group, potentially targeted the mRNA of the gene encoding cell wall integrity and stress response component 1-like (Figure 4B). This suggested that MSTRG.2885.5 may act as an antisense lncRNA to participate in the regulation of the cell wall integrity signaling pathway during the developmental process of larval guts. However, additional work is required to verify the interactions between these antisense DElncRNAs and their sense mRNAs, as well as the underlying mechanisms.

### 3.4. DElncRNAs May Regulate the Developmental Process of Larval Guts via ceRNA Networks

The ceRNA mechanism suggests that any RNA molecules containing miRNA response elements (MREs), such as lncRNAs, mRNAs, circRNAs, and pseudogenes, are able to competitively bind to miRNAs, and thus, modulate the expression of downstream target genes at the post-transcriptional level [8,55,56]. Studies have shown that lncRNAs play a pivotal part in the regulation of the growth and development of various insects, such as *Drosophila* [14], *Bombyx mori* [57], and *Aedes aegypti* [58]. In this study, a complicated ceRNA regulatory network was observed to exist among 75 (155) DElncRNAs, seven (5) DEmiRNAs, and 334 (248) DEmRNAs in the Ac4 vs. Ac5 (Ac5 vs. Ac6) comparison group (Figure S1). These target DEmRNAs could be annotated to 10 vital signaling pathways relevant to growth and development, such as the Hippo, Wnt, mTOR, Hedgehog, and insulin signaling pathways (Figure 6). The results together indicated that the corresponding DElncRNAs were likely to modulate larval gut development by regulating these 10 signaling pathways via ceRNA networks. In addition, it was detected that the target DEmRNAs were engaged in 7 (8) cellular-immune pathways and five (8) humoral-immune pathways, such as melanogenesis, necroptosis, the Jak-STAT signaling pathway, and the Ras signaling pathway (Figure 7). This was indicative of the involvement of corresponding DElncRNAs in the modulation of immunity during the developmental process of larval guts.

In *A. mellifera*, ame-miR-6001-5p has been suggested to play a regulatory part in ecdysone secretion and caste differentiation [59,60]. Following a comprehensive investigation of expression profiles and ceRNA networks, Guo et al. [61,62] discovered that both DElncRNAs and DEcircRNAs may act as ceRNAs to competitively target ame-miR-6001-y and regulate the development of *A. m. ligustica* workers’ midguts. Recently, Fan et al. [30] detected the differential expression of ace-miR-6001-y during the developmental process of *A. c. cerana* worker larval guts and found that ace-miR-6001-y might play a regulatory part by targeting downstream genes via a ceRNA network. In the present study, 54 and 116 DElncRNAs in the Ac4 vs. Ac5 and Ac5 vs. Ac6 comparison groups, respectively, were observed to target ace-miR-6001-y (Figure S2). Interestingly, ace-miR-6001-y was targeted by several shared DElncRNAs, such as MSTRG.12483.1, MSTRG.5739.5, and XR_003697233.1. These results indicated that the corresponding DElncRNAs may absorb DEmiRNAs and further affect the expression of downstream target genes in a ceRNA-network-dependent manner. More recently, our group performed an investigation on the feeding-based RNAi of the lncRNA13164 from *A. c. cerana* worker larval guts infected by *A. apis*, and an effective knockdown of the expression level of lncRNA13164 further affected the expression of the *stk*, *e3ul*, and *or1* genes via ace-miR-4968, which is suggestive of the regulation of host immune responses to an *A. apis* invasion mediated by the lncRNA13164-ace-miR-4968-immune gene axis [63]. In the near future, we will explore the mechanisms of the DElncRNA/DEcircRNA-miR-6001-y-DEmRNA axis by following our established technical platforms [64].

## 4. Materials and Methods

### 4.1. Bee Larvae

*A. cerana* worker larvae were obtained from three colonies reared in the teaching apiary of the College of Animal Sciences (College of Bee Science) at Fujian Agriculture and Forestry University (119.2369° E, 26.08279° N), Fujian Province, Fuzhou City, China.

### 4.2. Sample Preparation, RNA-Seq, and Quality Control

The gut tissues of *A. c. cerana* worker larvae were prepared and subjected to an RNA isolation and strand-specific cDNA library-based RNA-seq. Briefly, (1) 3-day-old *A. c. cerana* larvae (*n* = 48) were planted in a 48-well cell culture plate and were fed an artificial diet (royal jelly 63%, sterile water 30%, honey 6%, yeast extract 1%); (2) the larvae in the group were reared in a chamber (35 ± 0.5 °C, RH 90%) (Shanghai Yiheng Scientific Instrument Co., Ltd., Shanghai, China); (3) the guts of 4-, 5-, and 6-day-old larvae (named the Ac4, Ac5, and Ac6 groups, respectively) were dissected using our established protocol [65], producing a total of 9 gut tissue samples for each group; (4) the total RNA in the gut samples from each group was extracted using the TRIzol method (Promega, Madison, WI, USA), the cDNA fragments were purified with QIAquick PCR Purification Kit (Qiagen, Venlo, The Netherlands), end-repaired, polyA added, and then sequenced on an Illumina HiSeqTM 4000 platform (Guangzhou Gene Denovo Biotechnology Co., Ltd., Guangzhou, China); and (5) the produced raw data were subjected to a quality control procedure to obtain high-quality clean reads [66], which were then used for the analyses performed in this study. There were three replicates in this experiment. The raw data were deposited in the NCBI SRA database and linked to the BioProject number PRJNA565611.

### 4.3. Source of sRNA-Seq Data

In another previous work [30], the gut tissues of 4-, 5-, and 6-day-old *A. c. cerana* worker larvae were prepared, and this was followed by total RNA isolation, cDNA library construction, sRNA-seq by utilizing the Illumina HiSeq 4000 platform, and the strict quality control of raw reads. This experiment was repeated in triplicate. The raw data are available in the NCBI SRA database under the BioProject number: PRJNA562787.

### 4.4. Expression Level Calculation and Differential Analysis of lncRNAs

Following the FPKM (fragments per kilobase of transcript per million mapped reads) method [67], the expression level of each of the identified lncRNAs was calculated, which can eliminate the influence of different transcript lengths and sequencing data amounts on the calculation of transcript expression levels.

The edgeR software (v.4.2) [68] was employed to screen DElncRNAs in the Ac4 vs. Ac5 and Ac5 vs. Ac6 comparison groups by following the criteria of |log_2_(fold change)| > 1 and *p* < 0.05 (corrected by the false discovery rate).

### 4.5. Analysis of Cis-Acting Effect of DElncRNAs

According to the described method by Liu et al. [69], neighboring genes, the protein-coding genes within 10 kb upstream and downstream of the DElncRNAs were screened on the basis of their genomic positions. Next, analyses of GO terms (http://www.geneontology.org/ (accessed on 20 June 2023)) and KEGG pathways (https://www.kegg.jp/ (accessed on 20 June 2023)) enriched by the neighboring genes of DElncRNAs were performed by related tools in the Omicshare platform (http://www.omicshare.com/tools/index.php/ (accessed on 20 June 2023)) using the default parameters.

### 4.6. Analysis of DElncRNAs as Antisense lncRNAs

Antisense lncRNAs have been shown to regulate the abundance of target mRNAs via lncRNA–mRNA interactions [33,35,70]. RNAplex software (version 2.6.3) [71] was used to predict the complementary pairing relationships between antisense DElncRNAs and the corresponding target mRNAs; the criteria were set as a thermodynamic structure with free energy. Then, the best complementary pairing relationships were predicted.

### 4.7. Investigation of DElncRNA-Involved ceRNA Regulatory Networks

A collaboration of three software programs, namely miRanda (v.3.3a) [72], RNAhybrid (v.2.1.2) + svm_light (v.6.01) [73,74], and TargetFind [75], were employed to predict the DEmiRNAs targeted by DElncRNAs as well as the DEmRNAs targeted by DEmiRNAs. Further, the DElncRNA–DEmiRNA–DEmRNA regulatory network was constructed based on the target-binding relationships and then visualized using the Cytoscape software, v.3.2.1 [76].

### 4.8. RT-qPCR Validation

To confirm the reliability of the RNA-seq datasets used in this work, five DElncRNAs were randomly chosen for RT-qPCR detection. Specific primers for these selected DElncRNAs were designed using the DNAMAN software (v.9.0.1.116) and then synthesized by Sangon Biotech (Shanghai) Co., Ltd. The *actin* gene (GenBank accession number: XM_017059068.2) was used as an internal reference (Table S9). The total RNA from the gut tissues of 4-, 5-, and 6-day-old larvae (*n* = 3) was isolated with the RNA extraction kit. The resulting cDNA was used as the template for the qPCR reaction. The reaction for DElncRNAs was under the following conditions: pre-denaturation at 95 °C for 1 min, denaturation at 95 °C for 15 s, and extension at 60 °C for 30 s, with a total of 40 cycles. The reaction system (20 μL) contained 10 μL of SYBR green dye, 1 μL of each upstream and downstream primer at a concentration of 1 μmol/L, 1 μL of the cDNA template, and 7 μL of sterile water. The relative expression of the above DElncRNAs was calculated using the 2^-ΔΔCt^ method [77]. According to the described method by Wan et al. [78] and Liu et al. [79], the data are shown as the means ± standard deviation (SD) and were subjected to Student’s *t*-test by using the Graph Prism 8 software (ns, *p* > 0.05; *, *p* < 0.05; **, *p* < 0.01; ***, *p* < 0.001).

### 4.9. Dual-Luciferase Assay

The mimic of ace-miR-6001-y (mimic-miR-6001-y) and the corresponding negative control mimic (mimic-negative control, mimic-NC) were designed and then synthesized by Shanghai Gemma Pharmaceutical Technology Co. Next, the potential binding sites between MSTRG.11294.1 and miR-6001-y as well as between ncbi_107992440 and miR-6001-y were predicted using the RNA hybrid software (v.2.1.2). Specific primers for the aforementioned binding sites were designed (Table S10), followed by the use of PCR amplification. The amplified fragments were then cloned into pmirGLO vectors and named pmirGLO-lnc11294.1-wt and pmirGLO-mRNA2440-wt. At the same time, the mutant sequences of the above binding sites were designed and synthesized, and then cloned into pmirGLO vectors and named pmirGLO-lnc11294.1-mut and pmirGLO-mRNA2440-mut. The bacterial fluids were sent to Sangon Biotech (Shangha) Co., Ltd. for Sanger sequencing, and the bacterial fluids that had been sequenced correctly were transferred to a fresh liquid LB medium. Plasmids were extracted using an Endotoxin Removal Plasmid Extraction Kit (Beijing Total Gold Biotechnology Co., Ltd., Beijing, China).

The HEK-293T cells were spread into 96-well cell culture plates and then placed into a 37 °C incubator for 24 h to reach a cell density of 90–95%. Cell transfection experiments were carried out by following the instructions for the Hieff Trans™ Liposomal Nucleic Acid Transfection Reagent (Shanghai Yeasen Biotechnology Co., Ltd., Shanghai, China), and 8 transfection groups were set up at the same time: a: mimic-miR-6001-y co-transfected with pmirGLO-lnc11294.1-wt; b: mimic-NC co-transfected with pmirGLO-lnc11294.1-wt; c: mimic-miR-6001-y co-transfected with pmirGLO-lnc11294.1-mut; d: mimic-NC co-transfected with pmirGLO-lnc11294.1-mut; e: mimic-miR-6001-y co-transfected with pmirGLO-mRNA2440-wt; f: mimic-NC co-transfected with pmirGLO-mRNA2440-wt; g: mimic-miR-6001-y co-transfected with pmirGLO-mRNA2440-mut; and h: mimic-NC co-transfected with pmirGLO-mRNA2440-mut. After the transfection was completed, the cell culture plates were incubated in a 37 °C incubator for 24 h. Further, the viability of firefly fluoresceinase and Renilla fluoresceinase was detected on a dual-luciferase assay reporter system (Promega, Madison, WI, USA) using a dual-luciferase detection kit (Shanghai Yeasen Biotech Co., Ltd.), and the relative expression folds were obtained by calculating the ratio of firefly fluoresceinase/Renilla fluoresceinase. This experiment was repeated in triplicate. Following the method described by Matsumura et al. [80] and Wang et al. [81], the data are shown as the means ± standard deviation (SD) and were subjected to Student’s *t*-test by using Graph Prism 8 software (ns, *p* > 0.05; *, *p* < 0.05; **, *p* < 0.01; ***, *p* < 0.001).

## 5. Conclusions

**Figure 9:**
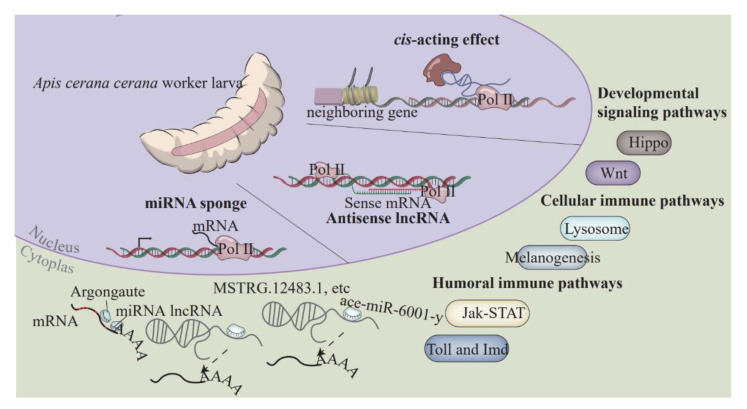
A hypothetical working model of diverse regulatory manners and potential roles of lncRNAs in the developmental process of *A. cerana* larval guts. Based on the results obtained in this study, differentially expressed lncRNAs (DElncRNAs) may be regulators in the larval gut development by affecting developmental signaling pathways as well as cellular- and humoral-immune pathways via cis-acting effect, antisense lncRNA, or competing endogenous RNA (ceRNA) network.

In a nutshell, the overall expression pattern of lncRNAs was dynamically changed during the developmental process of *A. c. cerana* worker larval guts. DElncRNAs were likely to participate in the modulation of the growth and development of larval guts in diverse manners, such as through the regulation of the transcription of neighboring genes, interactions with sense mRNAs, and the modulation of target gene expression via ceRNA networks; and through *cis*-actions, trans-actions, and ceRNA mechanisms affecting an array of sense-strand mRNAs and crucial pathways, including LOC108002698, LOC107997697, Wnt, and Hippo (Figure 9). The DElncRNA-miR-6001-y-DEmRNA axis may be an essential regulator in the development of larval guts. These findings reveal the role of lncRNA regulation during development. The targeting relationship was verified by a dual-luciferase reporter assay and provides a basis for the subsequent specific study of the mechanisms of action of lncRNAs.

## Data Availability

Raw data generated from RNA-seq are available in the NCBI SRA database under the BioProject numbers PRJNA565611 and PRJNA562787.

## Supplementary Materials

The supporting information can be downloaded at: https://www.mdpi.com/article/10.3390/ijms242015399/s1.
