# Supplementary material for: Diverse Regulatory Manners and Potential Roles of lncRNAs in the Developmental Process of Asian Honey Bee (Apis cerana) Larval Guts
Source: Int J Mol Sci. 2023 Oct 20;24(20):15399. doi: 10.3390/ijms242015399 (PMC10607868; doi:10.3390/ijms242015399)
Supplement: Supplementary file 1 [file ijms-24-15399-s001.zip › Supplementary Figures S1 and S2.pdf]

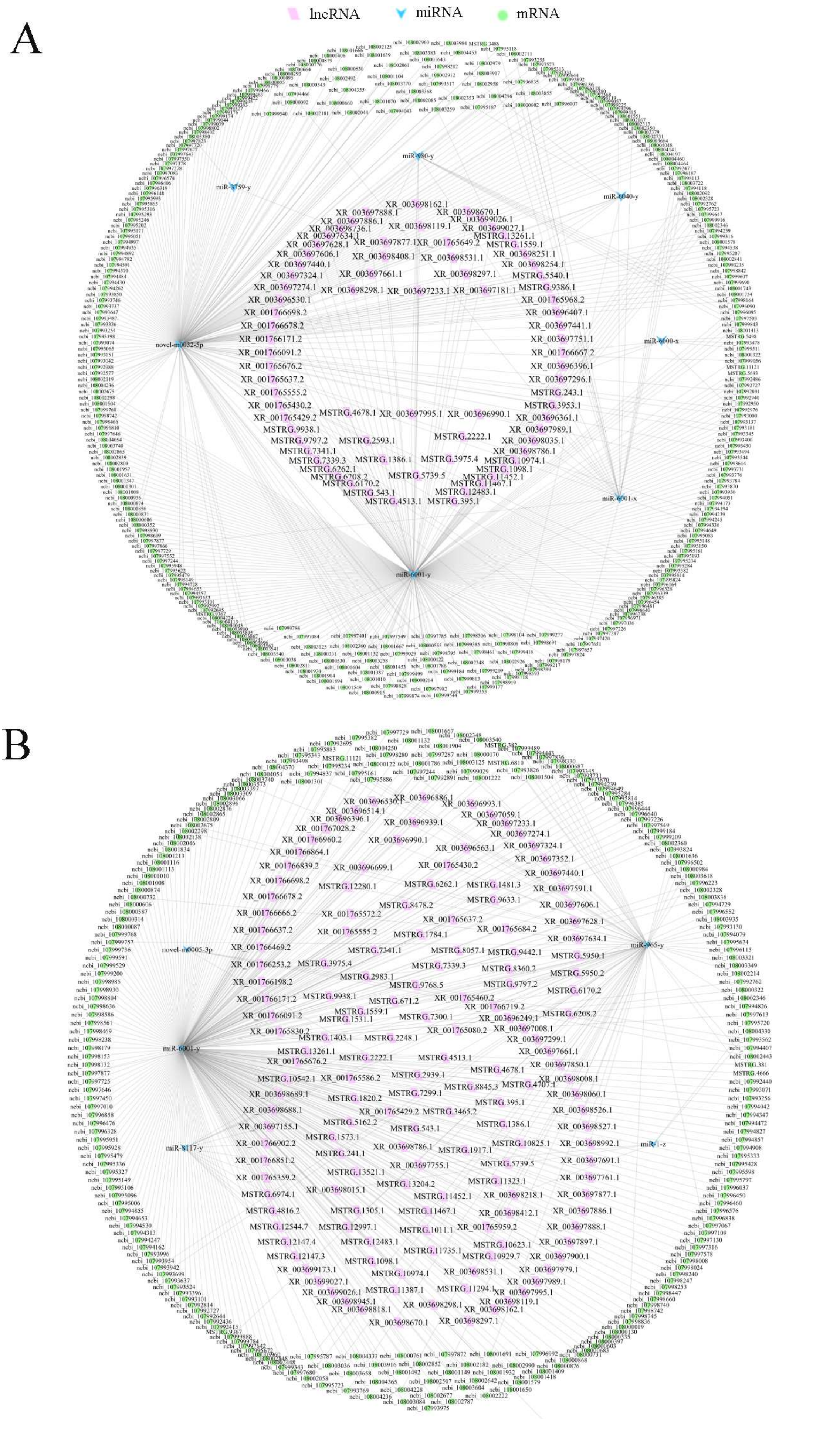
lncRNA
miRNA
mRNA
A
miR-980-y
miR-3759-y
miR-6040-y
novel-m0032-5p
miR-6000-x
miR-6001-x
miR-6001-y
B
novel-m0005-3p
miR-965-y
miR-6001-y
miR-8117-y
miR-1-z

Figure S1: ceRNA of DElnRNAs-DEmiRNAs-DEmRNAs in the Ac4 vs. Ac5 (A) and Ac5 vs. Ac6 (B) comparison group.

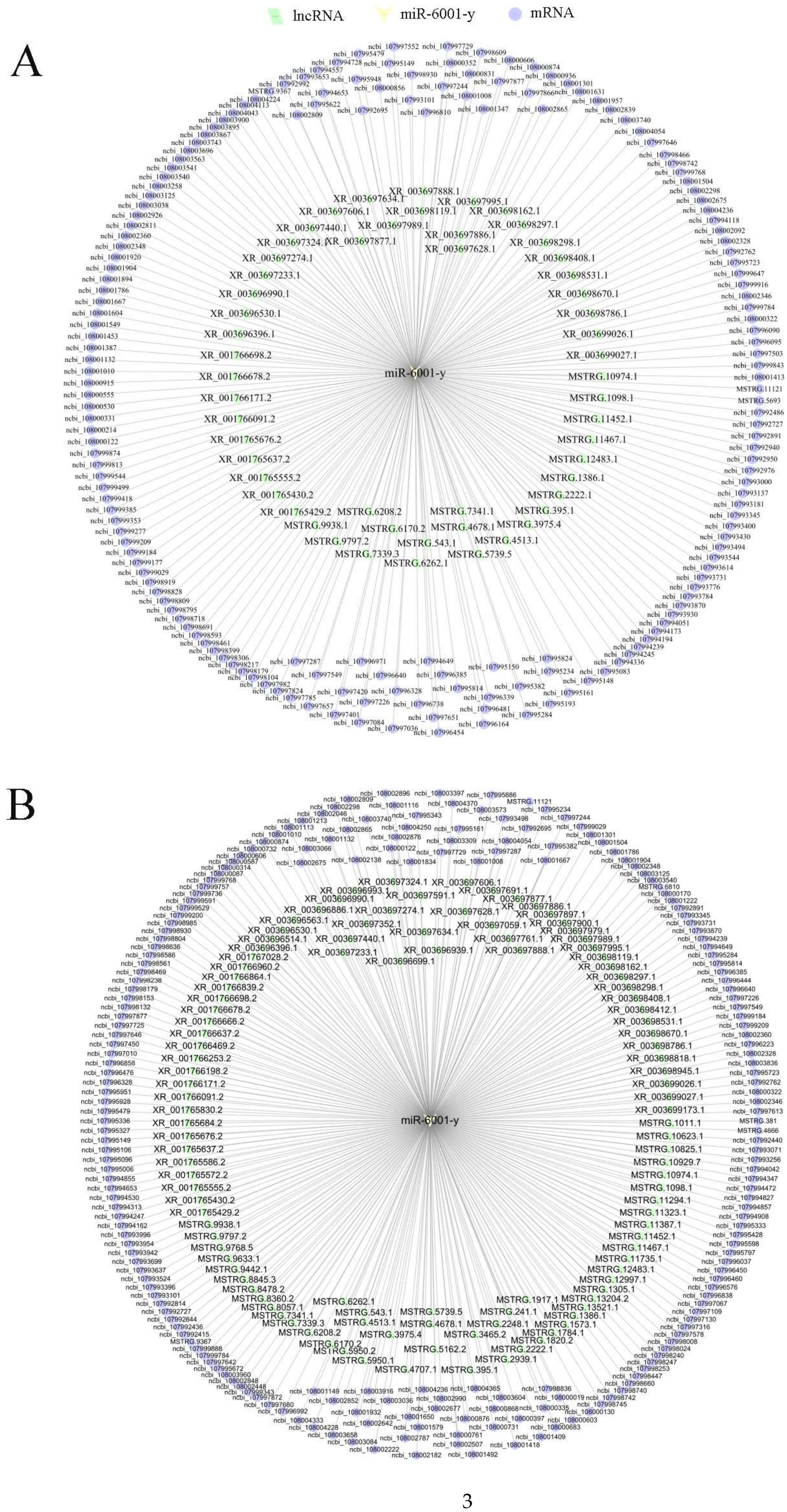
lncRNA
miR-6001-y
mRNA
A
miR-6001-y
B
miR-6001-y

Figure S2: Regulatory networks of DElncRNAs-miR-6001-y-DEmRNAs in Ac4 vs. Ac5 (A) and Ac5 vs. Ac6 (B) comparison group.
